# The eQTL-missense polymorphisms of *APOBEC3H* are associated with lung cancer risk in a Han Chinese population

**DOI:** 10.1038/srep14969

**Published:** 2015-10-13

**Authors:** Meng Zhu, Yuzhuo Wang, Cheng Wang, Wei Shen, Jia Liu, Liguo Geng, Yang Cheng, Juncheng Dai, Guangfu Jin, Hongxia Ma, Zhibin Hu, Hongbing Shen

**Affiliations:** 1 Department of Epidemiology and Biostatistics, Collaborative Innovation Center For Cancer Personalized Medicine, School of Public Health, Nanjing Medical University, Nanjing 211166, China; 2 Jiangsu Key Lab of Cancer Biomarkers, Prevention and Treatment, Collaborative Innovation Center of Cancer Medicine, Nanjing Medical University, Nanjing 211166, China

## Abstract

APOBEC (Apolipoprotein B mRNA editing enzyme, catalytic polypeptide-like) enzymes may involve in mutagenic processes in multiple cancer types, including lung cancer. APOBEC family of cytidine deaminases induces base substitutions with a stringent TCW motif, which is widespread in multiple human cancers. We hypothesized that common missense variants in coding regions of *APOBEC* genes might damage the structure of proteins and modify lung cancer risk. To test this hypothesis, we systematically screened predicted deleterious polymorphisms in the exon regions of 10 *APOBEC* core genes (*APOBEC1, APOBEC2, APOBEC3A, APOBEC3B, APOBEC3C, APOBEC3D, APOBEC3F, APOBEC3G, APOBEC3H*, and *APOBEC4*) and evaluated them with a case-control study including 1200 cases and 1253 controls. We found that the T allele of rs139293 in exon 2 of *APOBEC3H* was significantly associated with decreased risk of lung cancer (odds ratio = 0.76, 95% confidence interval: 0.63–0.91). Similar inverse association of this variant was observed in subgroups. Further study showed that the T allele of rs139293 was associated with the altered expression of *APOBEC3H* and *APOBEC3C* and that the two genes were co-expressed in both tumor and adjacent normal tissues. These results indicate that genetic variants in *APOBEC3H* may contribute to lung cancer susceptibility in Chinese population.

Lung cancer is the leading cause of cancer-related death, leading to 1.6 million deaths worldwide in 2012^1^. Although cigarette smoking is the major cause of lung cancer, genetic factors may also play a critical role in lung cancer risk. Recent genome-wide association studies (GWAS) have identified numerous loci related to lung cancer risk^2^,^3^,^4^,^5^,^6^,^7^,^8^,^9^,^10^,^11^. However, because of the stringent screening criteria of GWAS, these loci account for only a small fraction of familial risk of lung cancer. Moreover, limited by the number of sites in the array, many single nucleotide polymorphisms (SNPs) in key genes were not included in reported GWAS studies. An effort on candidate gene strategies might help to uncover the missing heritability^12^.

The mutation patterns of cancer genome are the aggregate outcome of multiple mutational processes leaving a characteristic mutational signature determined by the mechanisms of DNA damage and repair. APOBEC belongs to the family of cytidine deaminases and functions in RNA editing, humoral or innate immunity and DNA demethylation by catalyzing deamination of cytidine to uracil in single-stranded DNA (ssDNA) or RNA^13^,^14^,^15^. It was recently proposed to play an important role in generating particular genome-wide mutational signatures and induce clusters of localized hypermutation in human cancer genomes, named “kataegis”^16^,^17^. The study of *APOBEC* signature mutation based on whole-exome sequence data from The Cancer Genome Atlas (TCGA) suggests that cytosine deamination catalyzed by APOBEC enzymes is a mutagenic mechanism in multiple cancer types, including lung cancer^18^,^19^. Results from next-generation sequencing further showed that APOBECs induced base substitutions in tumor genomes with a stringent TCW motif (where W corresponds to either A or T), and this pattern was widespread in multiple human cancers^20^. Moreover, higher expression of *APOBEC3B* was identified significantly associated with the increased *APOBEC* signature mutations in lung cancer^21^,^22^.

However, even though the role of *APOBEC* in lung cancer genome has been identified, the association between genetic variants of *APOBEC* genes and susceptibility of lung cancer is still unknown. Here, we systematically screened common missense variants with predicted damaging effects in the exon regions of 10 *APOBEC* core genes (*APOBEC1, APOBEC2, APOBEC3A, APOBEC3B, APOBEC3C, APOBEC3D, APOBEC3F, APOBEC3G, APOBEC3H,* and *APOBEC4*) and conducted a case-control study including 1,200 cases and 1,253 controls to investigate the associations between these variants and lung cancer risk.

## Results

As shown in Table 1, the distributions of age and gender between the two groups were comparable. The proportion of smokers was significantly higher in lung cancer cases than those in controls (61.34% vs. 48.36%).

Genotyping rates for rs10911390, rs139293 and rs139299 were 100%, 98.12% and 99.96%, respectively. The observed genotype frequencies for these SNPs were in agreement with Hardy-Weinberg equilibrium in controls (Table 2). The genotype distributions of these three variants between cases and controls are shown in Table 2. The T allele of rs139293 was significantly associated with decreased risk of lung cancer with a per-allele adjusted odds ratio (OR) of 0.76 (95% confidence interval (95% CI): 0.63–0.91, *P* = 0.002). However, no significant associations were observed for the remaining two SNPs (rs10911390 and rs139299). To further characterize the association of rs139293 and lung cancer risk, stratified analysis was performed by age, sex, smoking status, smoking levels and histological types. The associations remained significant in the older subjects, never smokers, patients with squamous cell carcinoma and both males and females. However, no significant heterogeneity was found between any subgroups (Table 3).

As stated in the strategies of SNP selection, rs139293 was predicted to be as “probably damaging” in PolyPhen2 database. The SNP may contribute to the development of lung cancer by affecting the structure and function of APOBEC3H or it may also act as proxy of multiple rare variants. Dense fine-mapping of known regions usually identified novel functional rare variants^23^,^24^. To further evaluate the rare variants in the identified region, we annotated all rare SNPs located at the exons of *APOBEC3H* and in linkage disequilibrium with rs139293 (D’ = 1). Three rare variants were identified and all predicted to be deleterious according to SIFT or Polyphen2 database (Supplementary Table S2).

**Figure 1:**
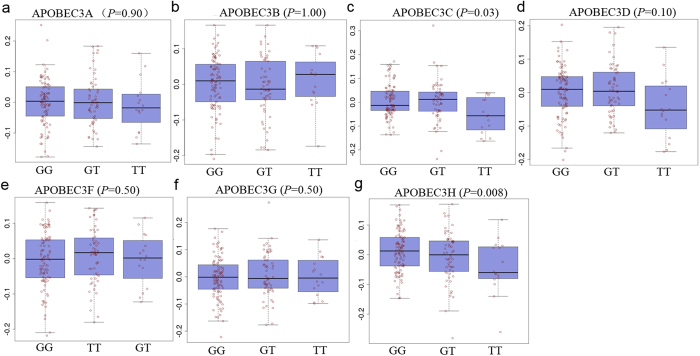
SNP rs139293 as a possible eQTL for *APOBEC* genes. The genotype of rs139293 and expression of *APOBEC* genes in blood were searched based on the public database GTEx Portal (**a–g**). The results show rs139293 were significantly correlated with the expression of *APOBEC3C* (**c**) and *APOBEC3H* (**g**).

In addition to impact the structure or function of protein directly, the identified SNP rs139293, based on the ENCODE and UCSC databases, was located at a regulatory element tagged by an active enhancer, histone H3K27Ac. The region was also a DNase I hypersensitive site and could bind multiple transcription factors (Supplementary Fig. S1). This indicated that rs139293 was also probably involved in the regulation of *APOBEC3H* expression. To validate this, we retrieved the genotype of rs139293 and expression of *APOBEC3H* in blood based on the public database GTEx Portal^25^. As Fig. 1 shows, the T allele of rs139293 was correlated with the reduced expression of *APOBEC3H* (*P* = 0.008). In consideration of distal effect of enhancer through chromatin interaction, we also analyzed the other 6 genes on the same chromosome (*APOBEC3A, APOBEC3B, APOBEC3C, APOBEC3D, APOBEC3F* and *APOBEC3G*). As Fig. 1 shows, the rs139293 was also an expression quantitative trait loci (eQTL) SNP for *APOBEC3C* (*P* = 0.03). To support the results, we found that the *APOBE3C* and *APOBEC3H* were co-expressed in both lung tumor and adjacent normal tissues based on TCGA database (ρ = 0.27, *P* = 5.51 × 10^−18^ in tumor tissues; ρ = 0.59, *P* = 1.60 × 10^−11^ in adjacent normal tissues) (Supplementary Fig. S2).

## Discussion

In this study, we systematically evaluated the association of common missense genetic variants in exons of core *APOBEC* genes and lung cancer risk in a case-control study including 1,200 cases and 1,253 healthy controls. And finally we found that the T allele of rs139293 was significantly associated with decreased risk of lung cancer in Chinese population. The SNP was predicted to be “probably damaging” according to Polyphen2 database. Further study showed that rs139293 was located at a regulatory region and was correlated with the expression of *APOBEC3H* and *APOBEC3C*.

Previous studies have shown that germline copy number polymorphism involving *APOBEC3A* and *APOBEC3B* (A3B^del^) are associated with a modest increased risk of breast cancer^26^. And breast cancers in carriers of the deletion show more mutations of the putative APOBEC-dependent genome-wide signatures than cancers in non-carriers^27^. Further mechanism study showed the A3B^del^ was associated with immune activation rather than cell proliferation which contribute to hypermutation^28^. There were few studies concerning the role of polymorphisms in *APOBEC3H* and cancer risk. However, the nature polymorphisms in human *APOBEC3H* have been associated with the stability and activity of the APOBEC3H protein when resistance to HIV-1 infection^29^.

The rs139293 variant was located at exon 2 of *APOBEC3H* and resulted in the amino acid substitution from arginine (Arg) to leucine (Leu) at codon 18. The variant was predicted to be deleterious on the structure and function of protein. Rare SNPs were more likely to have dramatic functional consequences but usually challenging to find^30^. Systematic annotation of rare SNPs in exons of *APOBEC3H* identified other three missense SNPs in linkage disequilibrium with rs139293 and all were predicted to be deleterious. Based on GTEx database, we found subjects with T allele of rs139293 had lower expression levels of *APOBEC3C* and *APOBEC3H* and this was further supported by the co-expression of these two genes in lung tumor and adjacent normal tissues. According to these results, we suggested that rs139293 was probably associated with reduced lung cancer risk by destroying the structure of APOBEC3H and regulating the expression of *APOBEC3C* and *APOBEC3H*.

To explore the possible mechanisms of rs139293 regulating the expression of *APOBEC3C* and *APOBEC3H*, we further annotated the SNP using ENCODE database (https://www.encodeproject.org/). We found the identified region can bind many transcription factors, including CTCF, EBF, MXI1, PAX5, RFX5, RUNX3, SIN3A, SMC3, TAF1 and WRNIP1^31^. Variants in transcription factor binding sites have been reported to be associated with cancer susceptibility^32^. This indicated that the region around rs139293 may play an important role in transcription regulation. More interestingly, we observed a chromatin interaction between chr22:39408338–39410119 and chr22:39493984–39496740 (hg19) in K562 cell line. The former located at the promoter of *APOBEC3C* and the later included the enhancer element around rs139293. The similar pattern of DNase I in exon colocalize with promoters had been reported previously^33^. These results suggested that the region around rs139293 was a transcriptional regulatory element and probably implicated in the regulation of *APOBEC3H* expression directly and *APOBEC3C* expression through chromatin interaction.

In summary, this study investigated the association of common missense genetic variants in *APOBECs* and lung cancer risk in a Chinese population. We found the missense SNP rs139293 of the *APOBEC3H* gene may modify the risk of lung cancer. However, our result is very preliminary, and the sample size is only moderate. Further independent studies incorporating functional evaluations are warranted to confirm the association and clarify the potential biological mechanisms of these polymorphisms in lung cancer risk.

## Materials and Methods

### Ethics statement

The study was performed in accordance with guidelines outlined in the Declaration of Helsinki and it was approved by the institutional review board of Nanjing Medical University (FWA00001501). The design and procedure of this study involving human participants were described in a research protocol. Written informed consent was obtained from every participant before the commencement of the study.

### Study population

All of the patients were histopathologically or cytologically confirmed as lung cancer by at least two pathologists. And those with a history of other cancers and ever received radiotherapy or chemotherapy were excluded from this study. A total of 1,200 cases recruited from the Cancer Hospital of Jiangsu Province and the First Affiliated Hospital of Nanjing Medical University between 2003 and 2009 were included in this study. All of the controls were randomly selected from individuals participating in a community based noncommunicable diseases screening program in Jiangsu Province during the same time period. Finally, 1,253 cancer-free controls frequency matched to the cases by age and sex were enrolled in this study. After provided with a written informed consent, we drawn 5 ml venous blood sample from each participant and took a face to face interview concerning demographic data (e.g. age and sex) and exposure information (e.g. smoking status). Current smokers were defined as those who had smoked one cigarette per day for >1 year; smokers who had quit smoking for >1 year were defined as former smokers; all others were classified as never smokers. Pack-years of smoking [(cigarettes per day/20) × smoking years] were calculated to measure smoking dose. In addition, smokers were divided into light and heavy smokers according to the threshold of 25 pack-years.

### SNP selection and genotyping

In this study, we mainly evaluated common missense variants in the coding region and focused on those predicted to have a damaging effect on the structure of proteins. SNPs in the exon regions of *APOBEC1, APOBEC2, APOBEC3A, APOBEC3B, APOBEC3C, APOBEC3D, APOBEC3F, APOBEC3G, APOBEC3H,* and *APOBEC4* were extracted from the 1000 Genomes database (the Phase I integrated variant set release V3, http://browser.1000genomes.org/index.html) and annotated by ANNOVAR (http://www.openbioinformatics.org/annovar/). SIFT (http://sift.jcvi.org/) and PolyPhen-2 (http://genetics.bwh.harvard.edu/pph2/) were further used to annotate the predicted function of these SNPs. SNPs meeting the following criteria were included in our study: (i) having a minor allele frequency (MAF) ≥5% in Chinese population; (ii) being a missense mutation in exon region; (iii) being predicted to have deleterious effects by SIFT and/or PloyPhen-2; and (iv) keeping only one SNP when multiple SNPs were in strong linkage disequilibrium (r^2^ ≥ 0.8). As a result, 278 SNPs with MAF > = 5% were further annotated by ANNOVAR, and among them 24 were located at exon regions (2 SNPs in *APOBEC1*, 2 SNPs in *APOBEC2*, 1 SNP in *APOBEC3A*, 3 SNPs in *APOBEC3B*, 1 SNP in *APOBEC3F*, 2 SNPs in *APOBEC3G*, 6 SNPs in *APOBEC3H*, 7 SNPs in *APOBEC4*). Fifteen of the 24 SNPs were missense and could result in amino acid change (Supplementary Table S1). However, only four were predicted to have a deleterious phenotypic effect, rs139293 (c.53G > T) and rs139299 (c.363G > C) in *APOBEC3H*, rs10911390 (c.1033G > A) and rs16861394 (c.224C > T) in *APOBEC4*. Because rs10911390 was in strong linkage disequilibrium with rs16861394 (R^2^ = 1), only rs139293, rs139299 and rs10911390 were genotyped in our study (Table 4).

Genomic DNA was isolated from a leukocyte pellet by proteinase K digestion, followed by phenol-chloroform extraction and ethanol precipitation. The genotyping were performed using the TaqMan allelic discrimination assay on the ABI 7900 system (Applied Biosystems, Foster City, CA, USA) and called using the SDS 2.3 Allelic Discrimination Software (Applied Biosystems). The primers and probes are available upon request. A series of methods were used to control the quality of genotyping: (i) genotyping was carried out without knowing the case or control status; (ii) two water controls were used in each plate as blank control; (iii) case and control samples were mixed on each plate; (iv) five percent of the samples were randomly selected for replicated genotyping.

### Public database

GTEx Portal was used to calculate the association of SNPs and gene expression in blood (http://www.gtexportal.org/home/). The Expectation-Maximization (RSEM) normalized read counts of *APOBEC* genes in lung tumors and adjacent normal tissues were downloaded from TCGA on date 07/08/2014. Transcription regulation were annotated using ENCODE database (https://www.encodeproject.org/) and UCSC genome browser (https://genome.ucsc.edu/cgi-bin/hgGateway).

### Statistical analysis

Deviation of genotype distribution for each SNP from the Hardy-Weinberg equilibrium was tested by a goodness-of-fit *χ*^2^. Student’s-t test for continuous variables and *χ*^2^ test for categorical variables were applied for analyzing distribution differences of demographic characteristics and genotypes between cases and controls. Correlation analysis was used to evaluate the co-expression of genes after log transformation. The association between SNPs and lung cancer risk was measured by odds ratios (ORs) and 95% confidence intervals (95% CI) using logistic regression with adjustment of age, sex and pack-years of smoking when appropriate. The tests above were two-sided and results were considered significant when *P* < 0.05. We used the *χ*^2^- based Q-test to test the heterogeneity from corresponding subgroups and the heterogeneity was considered significant when *P* < 0.10. All analyses were performed using R software (version 3.1.1).

## Additional Information

**How to cite this article**: Zhu, M. *et al.* The eQTL-missense polymorphisms of *APOBEC3H* are associated with lung cancer risk in a Han Chinese population. *Sci. Rep.* **5**, 14969; doi: 10.1038/srep14969 (2015).

## Supplementary Material

Supplementary Information

## Figures and Tables

**Table 1:** Demographic characteristics of the subjects in this study.

Variables	Case (*n* = 1,200)	Control (*n* = 1,253)	*P*-value
Age (Mean ± S.D.)^a^	60.99 ± 10.20	61.23 ± 10.82	0.569
<60	533 (44.42)	549 (43.81)	0.764
≥60	667 (55.58)	704(56.19)	
Gender			
Male	849 (70.75)	853 (68.08)	0.151
Female	351 (29.25)	400 (31.92)	
Smoking Status			<0.001
Current	569 (47.42)	553(44.13)	
Former	167 (13.92)	53 (4.23)	
Never	464 (38.66)	647 (51.64)	
Smoking Levels (Mean ± S.D.)^a^	41.33 ± 27.95	29.94 ± 20.61	<0.001
≤25 (Pack-years)	227 (30.84)	290 (47.85)	<0.001
>25 (Pack-years)	509 (69.16)	316 (52.15)	
Histology			
Squamous cell carcinoma	427 (35.58)		
Adenocarcinoma	773 (64.42)		

^a^Mean ± standard deviation in case and control groups respectively.

**Table 2:** Association of variants in *APOBEC* genes with lung cancer risk.

Variant ID	Cases^a^	Controls^a^	MAF^b^		HWE^c^	Crude OR (95% CI)	Adjusted OR (95% CI)^d^	*P*^d^
			Cases	Controls				
rs10911390	1053/144/3	1108/142/3	0.06	0.06	0.797	1.06 (0.84–1.35)	1.12 (0.88–1.42)	0.371
rs139293	957/206/20	912/297/15	0.10	0.13	0.108	0.75 (0.63–0.90)	0.76 (0.63–0.91)	0.002
rs139299	559/522/118	565/564/124	0.32	0.32	0.367	0.96 (0.85–1.09)	0.96 (0.85–1.09)	0.504

^a^Major homozygote/heterozygote/minor homozygote.

^b^MAF: Minor allele frequency.

^c^HWE: Hardy-weinberg equilibrium in controls.

^d^After adjusting for age, gender and pack-year of smoking.

**Table 3:** Stratified analysis on the associations of rs139293 in *APOBEC3H* with lung cancer risk.

Variables	Cases^a^	Controls^a^	OR (95% CI)^b^	*P*-value^b^	*P*-value^c^
Age (years)					
<60	422/94/8	402/125/7	0.78 (0.60–1.03)	0.080	0.723
≥60	535/112/12	510/172/8	0.73 (0.57–0.93)	0.012	
Sex					
Male	676/143/16	624/196/10	0.79 (0.64–0.98)	0.035	0.340
Femal	281/63/4	288/101/5	0.65 (0.47–0.92)	0.014	
Smoking states					
Never	374/77/6	460/164/8	0.65 (0.49–0.86)	0.003	0.122
Ever	583/129/14	452/133/7	0.87 (0.69–1.11)	0.273	
Smoking Levels					
≤25 (Pack-years)	177/42/5	214/65/2	1.02 (0.69–1.52)	0.906	0.393
>25 (Pack-years)	406/87/9	238/68/5	0.82 (0.60–1.11)	0.205	
Histological types					
Squamous carcinoma	352/63/5	912/297/15	0.65 (0.48–0.88)	0.005	0.163
Adenocarcinoma	605/143/15	912/297/15	0.84 (0.68–1.02)	0.082	

^a^Major homozygote/heterozygote/minor homozygote.

^b^After adjusting for age, gender and pack-year of smoking where appropriate assuming an additive genetic model.

^c^*P* for heterogeneity.

**Table 4:** Annotations of the selected variants in APOBEC genes.

Variant ID	Region	Position (hg19,bp)	Major/Minor	MAF^a^	Gene	Amino acid change	Location	Polyphen2^b^	SIFT^c^
rs10911390	1q25.3	183616884	C/T	0.08	*APOBEC4*	p.Val345Met	exon 2	B	D
rs139293	22q13.1	39496336	G/T	0.17	*APOBEC3H*	p.Arg18Leu	exon 2	D	T
rs139299	22q13.1	39497454	G/C	0.31	*APOBEC3H*	p.Lys121Asn	exon 3	B	D

^a^Minor allele frequency from CHB of 1000 Genomes.

^b^Possible impact of an amino acid substitution on the structure and function of a human protein based on PolyPhen 2 HDIV, D: Probably damaging; P: Possibly damaging; B: Benign.

^c^Possible impact of an amino acid substitution on the structure and function of a human protein based on SIFT, D: Deleterious; T: Tolerated.
