# Supplementary material for: The eQTL-missense polymorphisms of APOBEC3H are associated with lung cancer risk in a Han Chinese population
Source: Sci Rep. 2015 Oct 13;5:14969. doi: 10.1038/srep14969 (PMC4602211; doi:10.1038/srep14969)
Supplement: Supplementary Information [file srep14969-s1.pdf]

The eQTL-missense polymorphisms of *APOBEC3H* are associated with lung cancer risk in a Han Chinese population

Meng Zhu[1 ⊦], Yuzhuo Wang[1 ⊦], Cheng Wang[1 ⊦], Wei Shen[1], Jia Liu[1], Liguo Geng[1], Yang Cheng[1], Juncheng Dai[1,2], Guangfu Jin[1,2], Hongxia Ma[1,2], Zhibin Hu[1,2] and Hongbing Shen[1,2*]

Supplementary Figure S1. The identified SNP rs139293 in UCSC Genome Browser.

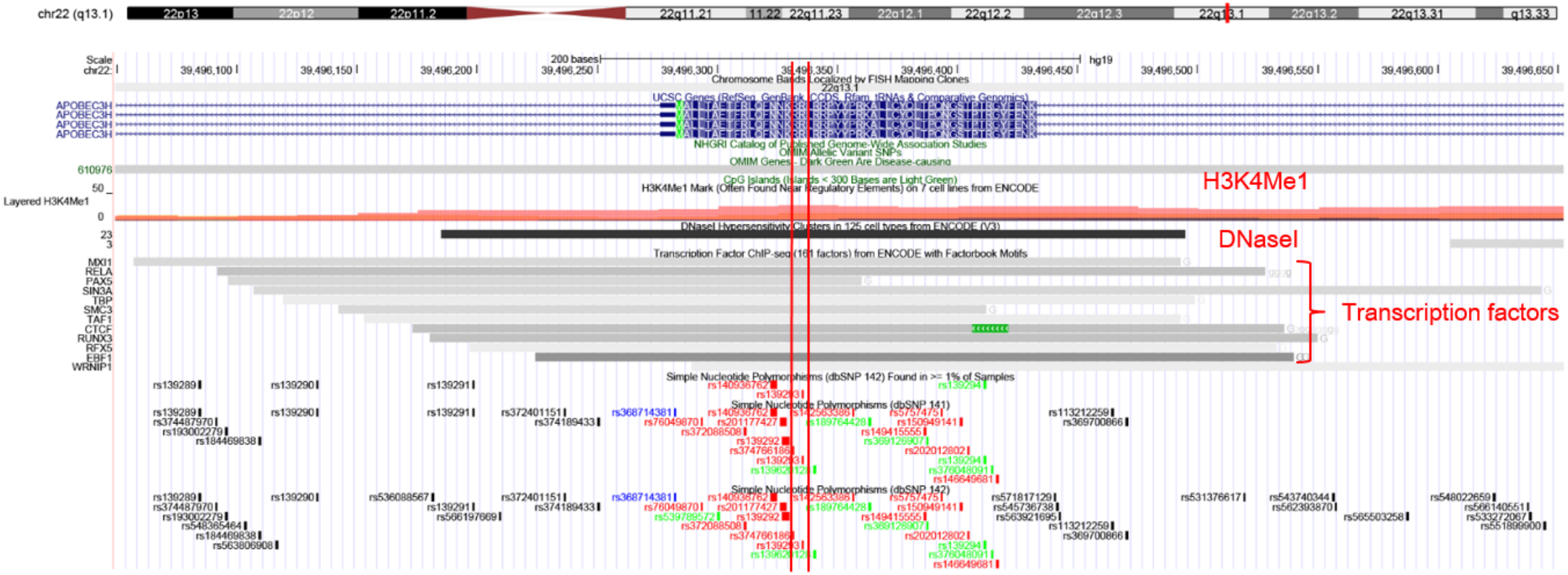

Supplementary Figure S2. The Co-expression of *APOBEC3C* and *APOBEC3H* based on TCGA data. a）Co-expression of *APOBEC3C* and *APOBEC3H* in lung tumour tissues. b) Co-expression of *APOBEC3C* and *APOBEC3H* in lung adjacent normal tissues. Spearman's rank correlation was used in this analysis.

a

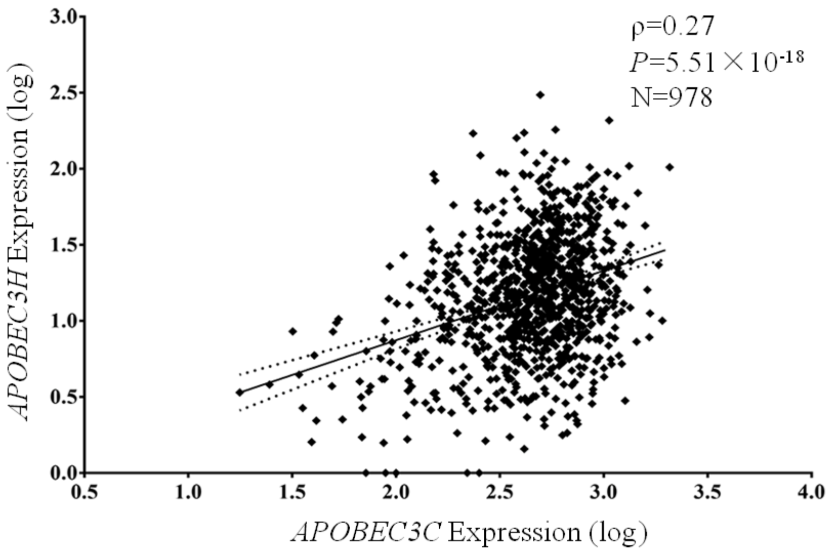

b

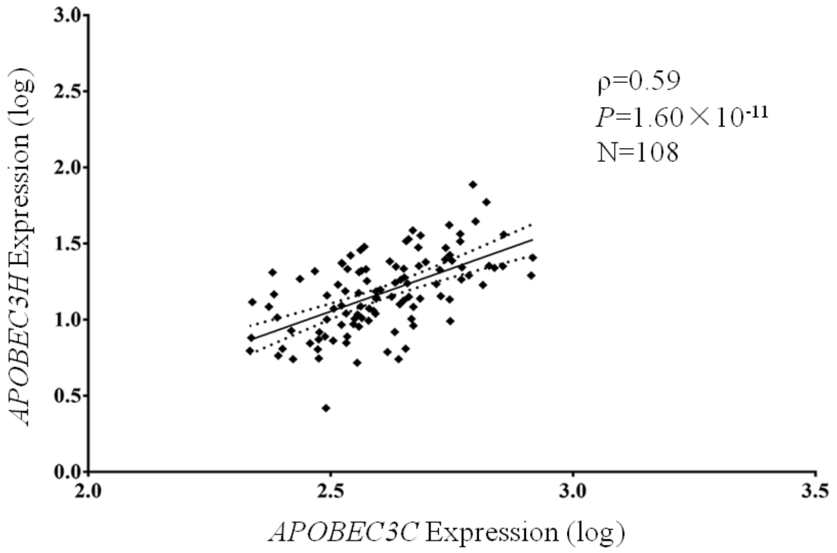

Supplementary Table S1. Variants located at the exons of *APOBEC* genes.

Variant ID	Region	Position (hg19,bp)	Major/Minor	MAF[a]	Gene	Function	SIFT[b]	Polyphen2[c]
**rs10911390**	**1q25.3**	**183616884**	**C/T**	**0.077**	***APOBEC4***	**missense**	**D**	**B**
rs1174657	1q25.3	183616926	T/C	0.175	*APOBEC4*	missense	T	B
rs10911391	1q25.3	183617094	G/A	0.077	*APOBEC4*	missense	T	B
rs1174658	1q25.3	183617105	A/G	0.175	*APOBEC4*	missense	T	B
rs10911392	1q25.3	183617311	T/A	0.077	*APOBEC4*	synonymy	.	.
rs12045762	1q25.3	183617413	T/C	0.077	*APOBEC4*	synonymy	.	.
**rs16861394**	**1q25.3**	**183617693**	**G/A**	**0.077**	***APOBEC4***	**missense**	**D**	**D**
rs2073014	6p21.1	41029109	T/C	0.237	*APOBEC2*	synonymy	.	.
rs2076472	6p21.2	41029342	T/C	0.247	*APOBEC2*	missense	T	B
rs10431309	12p13.31	7803646	G/A	0.320	*APOBEC1*	synonymy	.	.
rs2302515	12p13.31	7805236	G/C	0.392	*APOBEC1*	missense	.	.
rs72487648	22q13.1	39357581	C/T	0.284	*APOBEC3A*	synonymy	.	.
rs2076109	22q13.1	39381826	G/A	0.371	*APOBEC3B*	missense	.	.
rs2076111	22q13.1	39381999	C/T	0.387	*APOBEC3B*	synonymy	.	.
rs1065184	22q13.1	39387558	T/C	0.443	*APOBEC3B*	synonymy	.	.
rs5750728	22q13.1	39440149	T/C	0.289	*APOBEC3F*	missense	.	.
rs5757465	22q13.1	39477123	T/C	0.237	*APOBEC3G*	synonymy	.	.
rs8177832	22q13.1	39477566	A/G	0.062	*APOBEC3G*	missense	T	B
**rs139293**	**22q13.1**	**39496336**	**G/T**	**0.170**	***APOBEC3H***	**missense**	**T**	**D**
rs139294	22q13.1	39496412	G/C	0.345	*APOBEC3H*	synonymy	.	.
rs139297	22q13.1	39497404	G/C	0.309	*APOBEC3H*	missense	T	B
rs139298	22q13.1	39497452	A/G	0.309	*APOBEC3H*	missense	T	B
**rs139299**	**22q13.1**	**39497454**	**G/C**	**0.309**	***APOBEC3H***	**missense**	**D**	**B**
rs139302	22q13.1	39498038	G/C	0.340	*APOBEC3H*	missense	T	B

[a] Minor allele frequency of CHB from 1000 Genomes;

[b] Possible impact of an amino acid substitution on the structure and function of a human protein based on PolyPhen 2 HDIV, D: Probably damaging; P: Possibly damaging; B: Benign;

[c] Possible impact of an amino acid substitution on the structure and function of a human protein based on SIFT, D: Deleterious; T: Tolerated.

Supplementary Table S2. Rare variants in linkage disequilibrium with rs139293 in the exons of *APOBEC3H*.

Variant ID	Region	Position (hg19,bp)	Major/Minor	MAF[a]	Amino acid change	Location	Polyphen2[b]	SIFT[c]
rs112605061	22q13.1	39497412	C/A	0.005	p.Phe107Leu	exon 3	D	D
rs149415555	22q13.1	39496387	C/T	0.005	p.Thr35Met	exon 2	B	D
rs200886104	22q13.1	39499698	C/A	0.005	p.Ser183Tyr	exon 6	D	D

[a] Minor allele frequency of CHB from 1000 Genomes;

[b] Possible impact of an amino acid substitution on the structure and function of a human protein based on PolyPhen 2 HDIV, D: Probably damaging; P: Possibly damaging; B: Benign;

[c] Possible impact of an amino acid substitution on the structure and function of a human protein based on SIFT, D: Deleterious; T: Tolerated.
